# Sphingosine-1-Phosphate and the S1P_3_ Receptor Initiate Neuronal Retraction via RhoA/ROCK Associated with CRMP2 Phosphorylation

**DOI:** 10.3389/fnmol.2017.00317

**Published:** 2017-10-10

**Authors:** Serena Quarta, Maria Camprubí-Robles, Rüdiger Schweigreiter, Dusan Matusica, Rainer V. Haberberger, Richard L. Proia, Christine E. Bandtlow, Antonio Ferrer-Montiel, Michaela Kress

**Affiliations:** ^1^Division of Physiology, DPMP, Innsbruck Medical University, Innsbruck, Austria; ^2^Division of Neurobiochemistry, Biocenter, Innsbruck Medical University, Innsbruck, Austria; ^3^Anatomy & Histology, Centre for Neuroscience, College of Medicine and Public Health, Flinders University, Adelaide, SA, Australia; ^4^National Institute of Diabetes and Digestive and Kidney Diseases, Bethesda, MD, United States; ^5^Institute of Molecular and Cellular Biology, Miguel Hernández University, Elche, Spain

**Keywords:** S1P, S1P_3_, neurite outgrowth, axonal regeneration, CRMP2, sensory neurons, neurite retraction, neurite elongation

## Abstract

The bioactive lipid sphingosine-1-phosphate (S1P) is an important regulator in the nervous system. Here, we explored the role of S1P and its receptors *in vitro* and in preclinical models of peripheral nerve regeneration. Adult sensory neurons and motor neuron-like cells were exposed to S1P in an *in vitro* assay, and virtually all neurons responded with a rapid retraction of neurites and growth cone collapse which were associated with RhoA and ROCK activation. The S1P_1_ receptor agonist SEW2871 neither activated RhoA or neurite retraction, nor was S1P-induced neurite retraction mitigated in S1P_1_-deficient neurons. Depletion of S1P_3_ receptors however resulted in a dramatic inhibition of S1P-induced neurite retraction and was on the contrary associated with a significant elongation of neuronal processes in response to S1P. Opposing responses to S1P could be observed in the same neuron population, where S1P could activate S1P_1_ receptors to stimulate elongation or S1P_3_ receptors and retraction. S1P was, for the first time in sensory neurons, linked to the phosphorylation of collapsin response-mediated protein-2 (CRMP2), which was inhibited by ROCK inhibition. The improved sensory recovery after crush injury further supported the relevance of a critical role for S1P and receptors in fine-tuning axonal outgrowth in peripheral neurons.

## Introduction

Neuritogenesis is a complex event which involves a controlled balance between elongation and retraction of neuronal processes (da Silva and Dotti, 2002). The reorganization of the actin cytoskeleton, which is regulated by the opposing actions of the small GTPases Rho and Rac/Cdc42, is fundamental for this fine balance (Li et al., 2002). Rac/Cdc42 are required for the formation of lamellipodia or filopodia and thus neurite extension (Estrach et al., 2002), whereas Rho induces growth cone collapse and inhibition of neurite outgrowth (Nakamura et al., 2002). The growth promoting nerve growth factor (NGF) via its high affinity receptor TrkA activates sphingosine kinase type 1 (SphK1; Edsall et al., 1997), a kinase that phosphorylates sphingosine to produce sphingosine-1-phosphate (S1P). S1P is an important lipid mediator (Spiegel and Milstien, 2002) and can bind five specific G protein-coupled receptors (GPCRs), S1P_1_–S1P_5_. These receptors are enriched in the nervous system and couple to different G proteins, G_i_, G_q_ and G_12/13_, to transmit diverse downstream signaling. In particular, S1P_1_, S1P_2_ and S1P_3_ receptors are highly expressed in sensory neurons (Zhang et al., 2006; Mair et al., 2011; Kays et al., 2012; Camprubí-Robles et al., 2013; Li et al., 2015) and differentially regulate Rac and Rho. Signaling through S1P_1_ receptors enhances Rac-coupled lamellipodia extension (Rosenfeldt et al., 2001a), whereas S1P_2_ or S1P_3_ receptors stimulate Rho and suppress Rac activity, thereby inhibiting cell motility (Okamoto et al., 2000). In neuronal cell lines S1P rapidly induces neurite retraction and soma rounding with an EC50 as low as 1.5 nM, driven by Rho-dependent remodeling of the actin cytoskeleton (Postma et al., 1996). In contrast, the S1P_1,3,4,5_ receptor ligand FTY720 (fingolimod) enhances neurite outgrowth and alters growth cone morphology in primary cerebellar neurons. In accordance, FTY720 stimulates axon regeneration in mice after facial nerve axotomy through a signaling cascade involving S1P receptors, G_12/13_ G-proteins, RhoA-GTPases and the transcription factors SRF/MRTF (Anastasiadou and Knöll, 2016). However, it has been recently suggested that FTY720 rather acts as functional antagonist at S1P_1,3,4,5_ receptors (Hla and Brinkmann, 2011; Camm et al., 2014). Both, neurite extension as well as retraction can result from S1P action depending on the nature of S1P receptors that are expressed.

Therefore, we set out to explore the importance of S1P in peripheral nerve regeneration in a cellular model. Transgenic mice with ablations of SphK1, S1P_1_ or S1P_3_ receptors were used in combination with pharmacological tools. Together the data of the present study point towards a critical importance of S1P_3_ receptor for an overall deceleration of axonal growth, inducing fast neurite retraction through activation of Rho and Rho kinase (ROCK) and consequent phosphorylation of the collapsin response-mediated protein-2 (CRMP2). In accordance, S1P_3_ receptor ablation partially improved nerve regeneration in a preclinical model of peripheral nerve injury.

## Materials and Methods

### Ethics Statement

Experiments were performed according to National Austrian legal requirements and the recommendations of the IASP to minimize animal suffering (Zimmermann, 1983) with permission of the Austrian Bundesministerium für Wissenschaft, Forschung und Wirtschaft (BMWF-66.011/0102-WF/V/3b/2015).

### Mouse Strains and Surgical Procedure

Male wild-type (wt), S1P_3_^−/−^, SNS-S1P_1_^−/−^ and S1P_1_^fl/fl^ mice (age between 8 and 12 weeks) were used in all experiments. The mutated alleles were back crossed to C57BL/6J for at least 12 generations and littermates were used for controls (Mair et al., 2011). Animals were housed on a 12 h light/dark cycle and had free access to chow and water.

For induction of nerve crush injury mice were briefly anesthesizsed with Ketasol (Graeub, Innsbruck, Austria) and Xylasol (AniMedica) through intraperitoneal injection. After cutaneous incision, the sciatic nerve was exposed and crushed at mid-thigh level for 1 min by applying a standard force of 19 ± 1.8 N with a semi-electric feedback-controlled forceps as previously published (modified Bioseb Rodent pincher RP-1, Chaville Cedex, France; Quarta et al., 2014).

### Behavioral Testing

Animals were maintained in individual cages for the entire duration of the experimental paradigm of 15 days post lesion (dpl). Following nerve lesion, heat sensitivity was quantified by standard testing procedures (Andratsch et al., 2009; Quarta et al., 2014). Measurements were performed two times before injury and repeated at 1, 4, 8, 11, 15 dpl. Mice were allowed to habituate for 1 h before testing. Heat sensitivity was assessed using the Hargreaves test as previously published (Hargreaves et al., 1988), where the withdrawal response latency to an increasing heat stimulus (I.R. intensity = 51) was obtained with an automated algesiometer (Ugo Basile, Comerio, Italy).

### Culture of Primary Sensory Neurons

Adult mouse dorsal root ganglia (DRG) were dissected as previously published (Obreja et al., 2002; Agarwal et al., 2007). After dissection, ganglia were incubated in Liberase Blendzyme 1 (9 mg/100 ml DMEM, Roche, Vienna, Austria) for 60 min. After washing with PBS, 1× Trypsin-EDTA (Invitrogen, Vienna, Austria) was added for 15 min. Ganglia were washed with supplemented TNB™ medium (Biochrom, Berlin, Germany) containing L-glutamine (Invitrogen), streptomycin sulfate (Invitrogen), penicillin G sodium and Protein-Lipid-Komplex (Biochrom). The cell suspension obtained after mechanical dissociation was centrifuged at 500 rpm through a 3.5% BSA gradient (Sigma Aldrich, Vienna, Austria) for 10 min to remove cell debris. The pellet was resuspended in TNB™ medium and centrifuged for 5 min at 760 rpm. The neurons were plated on cover slips coated with poly-l-lysine (Sigma) and a top layer of laminin (10 μg/ml, Sigma). Neurons were cultured in TNB™ medium at 37°C in a 5% CO_2_ atmosphere for 24 h.

### Live Imaging of Sensory Neuronal Cultures

After 20 h, culture dishes were transferred to Axio Imager microscope (Carl Zeiss) with 20×, NA 0.30 objective. Images were recorded in phase contrast with a cooled CCD camera (SPOT; Diagnostic Instruments). Neurons were recorded at room temperature (RT) for 60 min with or without S1P or SEW2871 or FTY720-P at a concentration of 1 μM diluted in extracellular solution (ECS). Images at 0, 30 and 60 min were processed and compared as overlapped image for growth cone collapse and neurite retraction signs using MetaMorph image analysis (Meta Imaging Series 7.1, Version 7.1.6.0, Molecular Devices). Neurons were counted as elongating or retracting, if the majority of the neurites/neuron were respectively elongating or retracting, or as no effect, if there was no change in growth. For growth cone collapse assay, the distance between the growth cone position at 0, 30 and 60 min was observed and reported as difference. For inhibitor studies, neuronal cultures were pre-incubated with nontoxic concentrations of the specific inhibitor (C3-toxin) overnight or with Y27632 for 1 h before time lapse recordings. Retracted neurons were counted in one time-lapse recording frame before treatment and in the frame at 60 min after treatment. Control cultures were treated with equal volumes of vehicle which did not affect survival or outgrowth. During time lapse recording neurons were treated with 1 μM S1P for 60 min in the presence of the inhibitor.

### Immunocytochemistry and Quantification of Outgrowth of Sensory Neurons

Cells were fixed with 4% PFA for 20 min at RT. After permeabilization with 0.01% TX-100 (Pierce, Vienna, Austria) unspecific binding was blocked for 30 min with 10% normal goat serum (Sigma) in PBS. Cells were incubated with the first antibody (α-β-III-tubulin clone TuJ-1, 1:1000; R&D Systems, Vienna, Austria) for 1 h, washed 3× for 10 min with PBS and incubated with the appropriate secondary antibody for 30 min as previously published (Quarta et al., 2014), counterstained with 4′,6-diamidino-2-phenylindole (DAPI; 1:10.000; Sigma) and embedded in Mowiol (Calbiochem, Vienna, Austria). Secondary antibodies used were chicken α-mouse Alexa Fluo^®^ 594 or donkey α-mouse Alexa Fluo^®^ 488 (1:1000; Invitrogen) for fluorescence microcopy.

Digitized images of randomly chosen areas of the coverslip were taken using an Axio Imager microscope (Carl Zeiss) with 16×, NA 0.5 or 25×, NA 0.8 oil-immersion objective. Images were recorded with a cooled CCD camera (SPOT; Diagnostic Instruments). Experiments were performed in triplicates as minimum requirement. Only neurons that were clearly separated from neighboring cells were included for quantification. For the analysis of the percentage of neurite bearing cells, neurons with no visible process or with only filopodial formations were counted as negative. Cells showing at least one neurite with a total length of at least one cell diameter were counted as positive. Quantification of the total length was performed as previously (Quarta et al., 2014) using ImageJ software and NeuronJ plugin^1^ (Meijering et al., 2004).

### Culture of Motor Neuron-Like Cells

*Neuroblastoma x spinal cord motor neuron cells (NSC-34)* (kindly provided by Dr. Neil Cashman, University of Toronto, ON, Canada), were maintained in Dulbecco’s modified Eagle’s medium (DMEM) supplemented with 10% foetal bovine serum (FBS) and 1% penicillin/ streptomycin/glutamine solution (PSG) as previously described (Cashman et al., 1992). Cells were subcultured every 3–4 days. To slow the proliferation and enhance the differentiation, cells were grown to 60% confluence and the maintenance medium (DMEM, 10% FBS, 1% PSG) was exchanged for differentiating medium (1:1 DMEM plus Ham’s F12, 1% FBS, 1% PSG and 1% modified Eagle’s medium non-essential amino acids, NEAA), as previously described by Matusica et al. (2008). After 24–48 h culture, the medium was replaced with fresh differentiating medium and the survived cells were allowed to differentiate for up to ~7 days in the presence of 10 ng/ml BDNF and 1 ng/ml of CNTF. The media was refreshed every 2 days (Matusica et al., 2008).

### Live Imaging and Quantification of Outgrowth of Motor Neuron-Like Cells

Motor neuron-like cells (NSC34) were cultured in 24-well glass bottom plates coated with 0.015% poly-L-ornithine (Sigma) and differentiated as described previously (Matusica et al., 2008, 2016). Cells were seeded at a density of 3000 cells per well, differentiated over 48 h as described above, and vehicle or 1 μM of S1P (Sigma) was added to each well at the initiation time of image capture. Neurite outgrowth assays were performed on a FV1000 laser scanning confocal microscope (Olympus) equipped with a 5.1% CO_2_ perfused and temperature controlled (36.9°C) environmental control chamber (Solent Scientific). Image acquisition was performed on a 5 min interval over a period of 90 min, not including plate temperature calibration, using the multi-area time lapse (MATL) feature and the DIC filters engaged for the transmitted light image and 0.5 μm 20× lens correction. Four fields of cells were randomly imaged per well and analysis of neurite length and number of neurites/neuronal cluster (cells contained within the field of view with clearly identifiable processes) was performed on images using the NeuronJ plugin (ImageJ). All subsequent analysis was performed with Fiji (ImageJ) and Prism 7 (Graphpad, USA) software.

### Rho Activation Assay

Adult DRG neurons were cultured at a density of 2000 neurons/cm^2^ for 24 h and medium was replaced by ECS for 3 h before the experimental procedure. Cultures were treated for 10 min with S1P, FCS or SEW2871 as indicated. RhoA-GTP pulldown assays were performed as described previously (Schweigreiter et al., 2004). Briefly, neurons were washed twice with ice-cold TBS and lysed with ice-cold lysis buffer (500 μl; 50 mM Tris pH 7.2, 1% Triton X-100, 150 mM NaCl, 10 mM MgCl_2_, plus protease inhibitors). Lysed cells were scraped, collected into a pre-cooled Eppendorf tube and centrifuged at 14,000 rpm at 4°C for 5 min. The major part of the supernatant (about 450 μl) was added to 18 μl Rhotekin agarose beads (Millipore #14-383) and slowly rotated at 4°C for 50 min. A minor portion (50 μl) of the supernatant was used for assessment of total RhoA amount. Beads were washed three times with lysis buffer and elution and protein denaturation was done with 1× Laemmli buffer at 95°C for 5 min. Samples were electrophoresed in 15% polyacrylamide and proteins were transferred onto PVDF (polyvinylidene fluoride) membranes, which were blocked in 1% skim milk solution and incubated with anti-RhoA antibody (Santa Cruz #sc-418; 1:1000).

### Western Blotting

Lumbar sensory neurons were harvested from 6 wt mice per experiment and pooled for the following procedure. Sensory neurons were cultured for 24 h and growth factor-deprived in ECS (145 mM NaCl, 5 mM KCl, 2 mM CaCl_2_, 1 mM MgCl_2_, 10 mM HEPES, 10 mM D-Glucose; pH 7.3; Osm 305–308 mOsm/kg) before stimulation with S1P (1 μM) for 15, 30 and 60 min in the presence or not of the ROCK inhibitor Y27632 (10 μM). A pre-treatment of 1 h with Y27632 was performed. Non-treated cultures were used as controls. Neurons were harvested in freshly prepared ice-cold lysis buffer (50 mM Tris-HCl, 200 mM NaCl, 50 mM NaF, 1 mM EDTA, 20 mM β-Glycerophosphate, 1% Triton X-100) enriched with 1:30 phosphatase inhibitor complex (PIC, Sigma) and 1 mM phosphatase inhibitor sodium-ortho-vanadate (Sigma). Lysates were processed as described previously (Quarta et al., 2014) and protein content was measured with BCA™ Protein Assay kit (Thermo Scientific) according to the protocol. SDS-PAGE was conducted under standard denaturing conditions and 25 μg of protein were loaded per lane. For immunodetection, membranes were incubated with the specific antibodies diluted according to the manufacture instructions. Blots were visualized with enhanced chemiluminescence by using the SuperSignal West Pico Chemiluminescent Substrate (Thermo Scientific) and membranes scanned with LAS4000 luminescent imager (GE Healthcare). Quantification was performed using ImageJ software and relative values for phosphorylated proteins are related to the non-phosphorylated form. α-phospho-CRMP2 (Thr-555; 1:500; ECM Biosciences), α-CRMP2 (1:1000; Cell Signaling) and α-tubulin (1:2000; Sigma) were used as primary antibodies, and α-rabbit IgG (1:5000; Thermo Scientific), α-mouse IgG (1:10,000; Sigma) secondary antibody for detection.

### *In Situ* Hybridization

The protocol was modified from an earlier method from Obernosterer et al. (2007) and Camprubí-Robles et al. (2013). Cryostat sections of mouse spinal cord were fixed with 4% PFA, digested with proteinase K (2 μg/ml in 50 mM Tris buffer containing 5 mM EDTA), fixed again with 4% PFA, acetylated (triethanolamine/HCl/acetic anhydride) and incubated with prehybridization buffer (Roche). The S1P3 target mRNA was hybridized with 0.34 pmol of specific antisense double digoxigenin (DIG) labeled mRNA detection probes (S1P3, 5DigN/ACTGATG AGGAAGGCGATGTAT/3Dig_N) overnight at 55°C (Camprubí-Robles et al., 2013). Scrambled probes (Exiqon, Copenhagen, Denmark) served as negative control. Washing with saline sodium citrate buffer (SSC) was followed by blocking and incubation with alkaline phosphatase coupled anti-DIG antiserum (1:5000, Roche) for 1 h, followed by washing in levamisole buffer. Slides were incubated with BCIP/NBT (Roche) to visualize *S1p3* receptor mRNA, washed in DEPC-treated water, coverslipped in buffered glycerol and sealed. Images were taken using a brightfield microscope (BX50, Olympus).

### Isolation of Total RNA and Reverse Transcription

Differentiated NSC-34 cells treated with vehicle (Control) and S1P were harvested and stored in Trizol (Sigma) at −80°C. Samples were mechanically homogenized using a tissue lyser (Qiagen). Total RNA was isolated using a column based method (Zymo-Spin ICC Columns, Zymogen, Irvine, CA, USA). DNA contamination was removed by on-column DNA digestion. The concentration of total RNA was determined using standard photospectrometry (Nanodrop 2000, Thermo Scientific, Australia), quality of RNA was determined using a lab-on-chip system (Bioanalyzer, Agilent). Only samples with RNA integrity numbers (RIN) above seven were used for subsequent analysis. One microgram of total RNA was reverse transcribed into cDNA according to the manufacturer protocol (SuperscriptII, BioRad, Australia).

### qRT-PCR

Quantitative real-time polymerase chain reaction (qRT-PCR) analysis of the relative mRNA expression levels of differentiated NSC-34 cells treated with vehicle (Control) and S1P were performed using the StepOnePlus cycler (Life Technologies). TaqMan primers (Life Technologies) were used for the detection of peptidergic and non-peptidergic markers and molecules characteristic for mature nociceptive neurons. Hypoxanthine-guanine phosphoribosyl transferase (*HPRT*) was used as a reference gene. The efficiencies of all primer-pairs were determined by 1/5 to 1/625 dilutions in a qPCR and a satisfying efficiency was determined with Q-Gene (Simon, 2003). The primers and efficiencies are listed in Table 1. The final volume for qPCR was 20 μl of which 8 μl were H_2_O, 10 μl mastermix (Life Technologies), 1 μl assay-mix (Life Technologies) and 1 μl cDNA. Each q-RT-PCR was done in duplicate. The Ct values were determined for each product and normalized as pairwise comparisons against the Ct value of the reference gene. Subsequently the mean normalized expression (MNE) was calculated and differences in the expression determined (Simon, 2003).

**Table 1:** TaqMan primer assay.

Primer assay	Assay code	Amplicon length (bp)	Efficiency (R2)
Hprt1	Rn1527840_m1	64	0.995
S1pr3	Mm02620181_s1	120	0.997

### Compounds and Inhibitors

Factors used: 50 μM ABC294640 (Active Biochemicals Co., Limited; Hong Kong); 10 nM, 100 nM or 1 μM S1P (Sigma) dissolved in 0.1% methanol. Inhibitors and agonists: 0.5 μg/ml C3 toxin (Cytoskeleton, Inc.); 10 μM Y27632 (Calbiochem); 10 μM SEW2871 (Calbiochem); 1 μM FTY720-P (Echeion Biosciences).

### Experimental Design and Statistical Analysis

All experiments were performed in cell cultures/materials from at least three different animals, and numbers of cells are reported under each figure legend. Behavioral assays were performed in 10 or 11 male animals per genotype, as reported under the figure legend. For statistical analysis the GraphPad software package 6 or 7 was used and data are presented as mean ± SEM. Two-way repeated measurements ANOVA followed by Tukey *post hoc* test or Mann-Whitney U- *post hoc* test was used for multiple comparisons between groups for behavioral assays. One-way or two-way ANOVA followed by *post hoc* test was used for multiple comparisons between groups for *in vitro* experiments. Kruskal-Wallis test or Mann-Whitney U-test was calculated for interindividual comparisons for *in vitro* experiments. Data were analyzed by *χ*^2^-test for comparison of relative group sizes for *in vitro* experiments. Individual test are reported under each figure legends. Differences were considered statistically significant at *p* < 0.05.

## Results

### S1P Significantly Reduces Neurite Outgrowth of Adult DRG Neurons *in Vitro*

**Figure 1:**
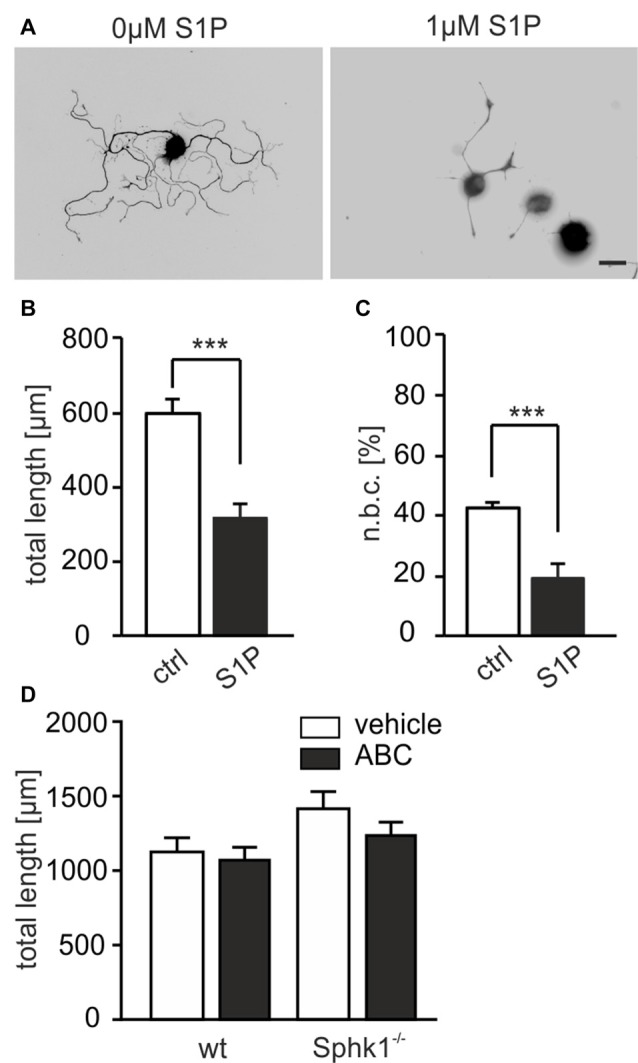
Sphingosine-1-phosphate (S1P) reduces neurite outgrowth of adult dorsal root ganglia (DRG) neurons *in vitro*. **(A)** Representative examples of DRG neuron cultures from wild-type (wt) mice under the effect of S1P. Scale bar = 30 μm. Neurons were cultivated with or without S1P (1 μM) for 24 h and stained with Tuj-1. **(B)** Neurite length of 24 h cultured wt neurons was significantly decreased upon S1P treatment (596.4 ± 41.48 μm for ctrl vs. 318.4 ± 35.28 μm for S1P-treated, ***p < 0.001, U-test; ctrl *n* = 209, S1P *n* = 129). **(C)** The percentage of neurite bearing cells in treated cultures was significantly reduced compared to untreated cultures (42.88 ± 1.82% for ctrl vs. 19.33 ± 4.93% for S1P-treated, ***p = 0.000391, *χ*^2^-test; ctrl *n* = 209, S1P *n* = 129). **(D)** Treatment for 24 h with SphKs inhibitor ABC294640 (ABC) did not induce any significant effect on the total neurite length of either wt or Sphk1^−/−^ DRG neurons (n.s., Kruskal-Wallis test, *p* = 0.092; 1071.03 ± 85.33 μm for wt ABC and 1235.60 ± 89.79 μm for Sphk1^−/−^ ABC; wt vehicle *n* = 337, ABC *n* = 291; Sphk1^−/−^ vehicle *n* = 279, ABC *n* = 279).

The involvement of S1P in neuronal outgrowth was first studied in short term cultures of adult DRG neurons as an *in vitro* model for peripheral nerve injury. Only 20% of adult DRG neurons had visible processes when neurons were grown in culture for 24 h with 1 μM S1P compared to the 43% of neurons which were treated with vehicle (Figures 1A,C; 42.88 ± 1.82% for ctrl vs. 19.33 ± 4.93% for S1P-treated, *p* = 0.000391, *χ*^2^-test). At the same time the total neurite length of neurons in S1P-treated cultures was significantly reduced after 24 h of S1P treatment (Figures 1A,B; 596.4 ± 41.48 μm for ctrl vs. 318.4 ± 35.28 μm for S1P-treated, *p* < 0.001, U-test). S1P synthesis was previously suggested to act downstream of the NOGO signaling pathway, which has a significant anti-regenerative potential (Kempf et al., 2014) and NGF signaling causes SphK1 translocation to the plasma membrane with subsequent activation of the S1P_1_ and S1P_2_ receptors (Toman et al., 2004). Thus, we first explored whether SphKs, involved in S1P production, were involved in the intrinsic extension of neuronal processes in adult sensory neurons. Neuron cultures were obtained from mice with a global null mutation of SphK1 (Sphk1^−/−^) and grown in the presence or absence of the SphK2 inhibitor ABC294640 (ABC; Gao et al., 2012). The inhibition of the activity of both SphK isoforms by knock-out and ABC treatment for 24 h did not exhibit any significant effect on neurite outgrowth (Figure 1D; *p* = 0.092, Kruskal-Wallis test), indicating that inhibition of SphK activity did not alter neurite outgrowth of sensory neurons in the absence of exogenous S1P. Together, these results suggest that S1P might serve a critical role in neurite outgrowth of sensory neurons, *in vitro*, and that autocrine S1P signaling appears not to be critically involved in the outgrowth of sensory neurons.

### Dose and Time-Dependent S1P-Induced Retraction of Adult DRG Neurons *in Vitro*

**Figure 2:**
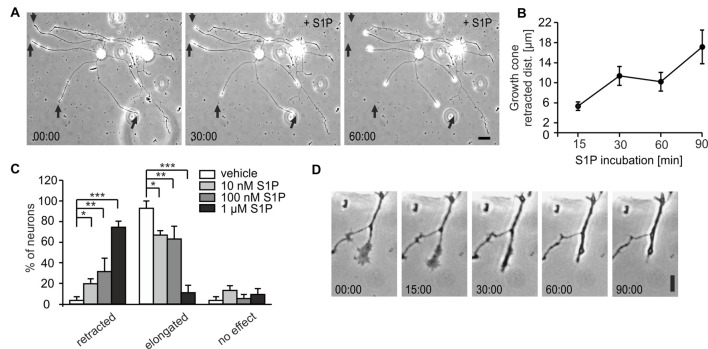
Dose and time-dependent S1P-induced retraction of adult DRG neurons *in vitro*. **(A)** Representative example of wt adult sensory neurons at 0, 30 and 60 min after S1P and the retractive effect on already existing neurites. Scale bar = 30 μm. **(B)** Growth cones of adult wt DRG neurons retracted within 15 min upon 1 μM S1P treatment. The distance covered by retracting growth cones was significantly higher at 90 min compared to 15 min (*p* = 0.0014, one-way ANOVA followed by Tukey *post hoc* test; *n* = 41). **(C)** S1P elicited a retractive effect in a dose-dependent manner, with nearly 80% of cells retracting and the number of elongating neurons decreasing after 1 μM S1P (*p* = 0.0001, *χ*^2^-test; vehicle *n* = 35, S1P 10 nM *n* = 65; 100 nM *n* = 65; 1 μM *n* = 30). **(D)** Representative example at higher magnification of growth cone collapse after S1P treatment. Scale bar 10 μm. *p < 0.05, **p < 0.01, ***p < 0.001.

Since we and others have previously reported S1P effects to occur on a fast time scale (Postma et al., 1996; Toman et al., 2004; Mair et al., 2011; Camprubí-Robles et al., 2013), we explored whether S1P affected neurons with already existing neurites and applied S1P for short durations to cultures in a live imaging set-up (15, 30, 60 and 90 min). In wt neurons, S1P induced a time-dependent retraction of neuronal processes which was visible after 15 min (Figures 2A,B) and increased with prolonged S1P exposure (Figure 2B). The percentage of neurons with retracting neurites was significantly increased in a dose-dependent manner: 10 nM S1P was already sufficient to induce a slight retraction, however more neurons were elongating than retracting their neurites, and nearly 80% of sensory neurons retracted their neurites in response to 1 μM S1P (Figures 2A,C; *p* = 0.0001, *χ*^2^-test). Similarly, the number of neurons which showed elongating neurites was profoundly decreased to 11.8 ± 7.15% upon 1 μM S1P (Figure 2C). The retraction in response to 1 μM S1P was accompanied by growth cone collapse visible at higher magnification (Figures 2B,D). These results revealed that physiologically relevant concentrations of S1P caused fast retraction of already existing neurites of adult sensory neurons.

### S1P_3_ Receptor Is Involved in the Retraction Adult DRG Neurons

**Figure 3:**
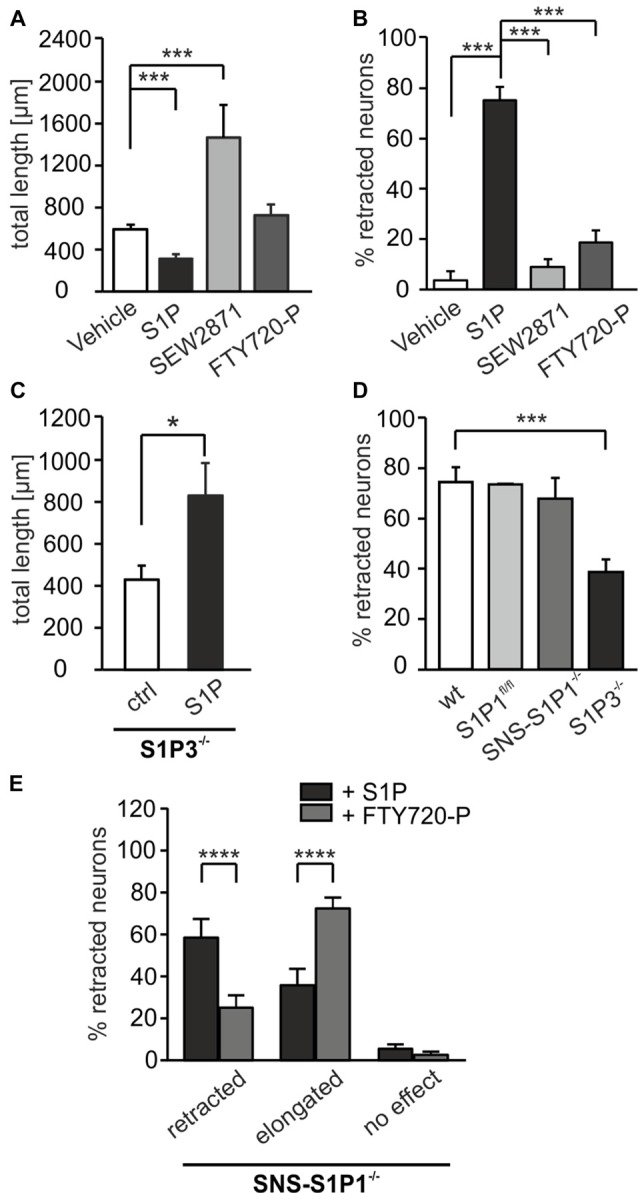
S1P_3_ receptor is involved in neurite retraction while S1P_1_ in elongation of adult DRG neurons. **(A)** S1P receptor agonists SEW2871, which activates S1P_1_, and FTY720-P, agonist for S1P_1,3,4,5_, produced a different effect compared to S1P. SEW2871 increased significantly the total neurite length, which was decreased in presence of S1P compared to untreated 24 h DRG cultures (***p < 0.001, U-test; ctrl *n* = 209, S1P *n* = 129, SEW2871 *n* = 124, FTY720-P *n* = 138). **(B)** The percentage of retracted neurons was significantly decreased upon 60 min SEW2871 or FTY720-P treatment compared to S1P-treated cultures (***p < 0.001, *χ*^2^-test; ctrl *n* = 27, S1P *n* = 76, SEW2871 *n* = 104, FTY720-P *n* = 87). **(C)** Adult sensory neurons from mice with global deletion of S1P_3_ receptor (S1P_3_^−\−^) showed a significant increase in total neurite length after 24 h S1P treatment (*p = 0.0432, U-test; ctrl *n* = 150, S1P *n* = 100). **(D)** 60 min of S1P treatment on already existing neurites exhibited a significant decrease in the number of retracted neurons in S1P_3_^−\−^ DRG cultures (***p < 0.001, *χ*^2^-test), in contrast, SNS-S1P_1_^−\−^ neurons showed similar effect to S1P of wt neurons (n.s., *χ*^2^-test; wt *n* = 76, S1P_1_^fl/fl^ *n* = 31, SNS-S1P_1_^−\−^ *n* = 110, S1P_3_^−\−^ *n* = 191). **(E)** Adult sensory neuron cultures from SNS-S1P_1_^−\−^ mice showed reduced retraction and enhanced elongation upon FTY720-P treatment (****p < 0.0001, *χ*^2^-test; S1P *n* = 155, FTY720-P *n* = 71).

Of the generally accepted five S1P receptors, S1P_1_, S1P_2_ and S1P_3_ subtypes are expressed in DRG neurons (Zhang et al., 2006; Mair et al., 2011; Camprubí-Robles et al., 2013). Out of those three receptor subtypes, Toman et al. (2004) showed that S1P_1_ receptor is involved in neurite extension, whereas S1P_2_ and S1P_3_ receptors have been associated, respectively, with processes retraction in PC12 cells (Toman et al., 2004) and cell rounding (Van Brocklyn et al., 1999). We first used SEW2871 to activate the S1P_1_ receptor, or FTY720-P, a ligand at S1P_1,3,4,5_ receptors. In contrast to S1P, both, SEW2871 and FTY720-P induced elongation of neurites (Figure 3A; *p* < 0.001, U-test), and increased the number of neurons with elongating neurites, rather than neurite retraction (Figure 3B; *p* < 0.001, *χ*^2^-test). It should be noted that DRG neurons do not express S1P_4_ or S1P_5_ and only a negligible fraction of neurons in our culture conditions express S1P_2_ receptor subtype (Camprubí-Robles et al., 2013). Since retraction was observed in more than 80% of DRG neurons upon 1 μM S1P, we hypothesized that S1P-induced neurite retraction could be mediated through activation of S1P_3_ receptors. To prove this hypothesis, DRG neuronal cultures from transgenic mouse lines with global or sensory neuron specific deletions of S1P receptors were exposed to S1P. In cultures from adult S1P_3_^−/−^ mice, the average total neurite length was significantly increased in the presence of S1P after 24 h (426.5 ± 69.32 μm vs. 825.4 ± 158.8 μm; *p* = 0.0432, U-test; Figure 3C). Moreover, in S1P_3_^−/−^ primary DRG cultures, the percentage of neurons that responded to S1P after 60 min with a retraction was significantly reduced compared to wt (74.49 ± 5.89% vs. 38.74 ± 4.98%; *p* < 0.001, *χ*^2^-test; Figure 3D). In contrast, a conditional, sensory neuron specific deletion of the S1P_1_ receptor subtype (SNS-S1P_1_^−/−^) did not produce a significant change in the percentage of retracted neurons compared to control cultures (Figure 3D). SNS-S1P_1_^−/−^ mice lack S1P_1_ mRNA expression and immunoreactivity specifically in DRG neurons (Mair et al., 2011). Sensory neuron cultures from SNS-S1P_1_^−/−^ mice showed reduced elongation upon S1P treatment, however, upon exposure to FTY720-P we observed a significant increase in the elongation compared to S1P treated cultures (Figure 3E; *p* < 0.0001, *χ*^2^-test). This puzzling result could be explained with the recently reported antagonistic effect of FTY720-P at S1P receptors (Hla and Brinkmann, 2011; Camm et al., 2014) and possible inhibition at S1P_3_ receptor subtype, since sensory neurons in culture express mainly S1P_1_ and S1P_3_ receptors, as mentioned above.

These observations support the concept that S1P binding to S1P_3_ receptor acts to facilitate neurite retraction of primary sensory neurons. In contrast, activation of S1P_1_ receptor may rather promote neurite elongation.

### S1P Induces Retraction via Rho/ROCK Activation and CRMP2 Phosphorylation

**Figure 4:**
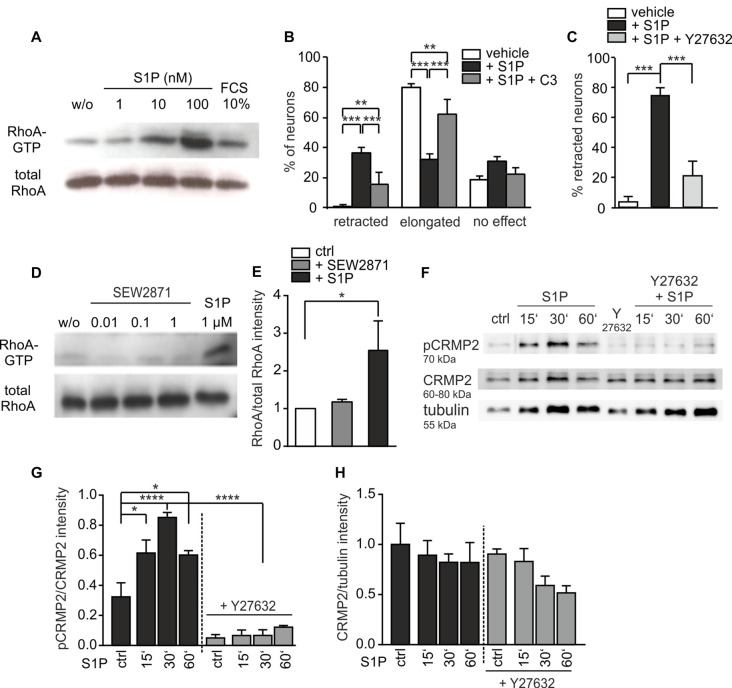
S1P induces retraction via Rho/ROCK activation and collapsin response-mediated protein-2 (CRMP2) phosphorylation. **(A)** RhoA activation was observed in a pull-down assay upon 10 min S1P treatment of adult DRG neurons cultured for 24 h. **(B)** C3-toxin, which inhibited Rho activation, reversed the S1P-dependent retraction decreasing the number of retracted neurons compared to S1P only -treated cultures (S1P vs. S1P + C3 retracted *p* = 0.0005, elongated *p* < 0.001, two sample *t*-test between percents; vehicle *n* = 77, S1P *n* = 108, S1P + C3 *n* = 106). Cultures were pre-treated overnight with C3-toxin and then treated with 1 μM S1P for 60 min in the presence of the toxin. **(C)** Treatment with Y27632 compound reversed the retraction effect of 1 μM S1P (*p* < 0.001, *χ*^2^-test; vehicle *n* = 27, S1P *n* = 76, S1P + Y27632 *n* = 134). Cultures were pre-treated for 1 h with Y27632 before S1P stimulation. **(D)** Different concentrations of SEW2871 after 10 min stimulation did not activate downstream RhoA in adult sensory neuronal cultures. **(E)** Quantification of RhoA levels obtained from cultures before and after 1 μM SEW2871 or 1 μM S1P treatment (ctrl vs. SEW2871, n.s. *p* = 0.5172; ctrl vs. S1P, *p = 0.019, Kruskal-Wallis test followed by Dunn’s *post hoc* test; *n* = 3). **(F)** Representative western blot for phospho-CRMP2 (pCRMP2 Thr-555) levels in wt DRG neuronal cultures before and after 15’, 30’ and 60’ S1P treatment (1 μM). **(G)** Quantification of pCRMP2 levels vs. CRMP2 obtained from treated and untreated cultures. Levels of total CRMP2 were similar in all samples. pCRMP2 levels increased significantly upon S1P stimulation, with a peak of CRMP2 phosphorylation/inactivity at 30 min (****p < 0.0001, one-way ANOVA followed by Tukey *post hoc* test; *n* = 3, ctrl vs. S1P 15’ *p* = 0.019, ctrl vs. S1P 30’ *p* < 0.0001, ctrl vs. S1P 60’ *p* = 0.027). These levels were significantly reduced, thus CRMP2 phosphorylation was reversed, upon Y27632 inhibitor (S1P 30’ vs. Y27632 + S1P 30’ *p* < 0.0001). **(H)** Total CRMP2 levels were similar in all samples and not significantly different compared to tubulin (*p* = 0.2478, one-way ANOVA; *n* = 3). *p < 0.05, **p < 0.01, ***p < 0.001, ****p < 0.0001.

S1P_3_ receptor activates members of the Rho GTPase family (Pyne and Pyne, 2000a, b; Sanchez and Hla, 2004; Obinata and Hla, 2012) which are known regulators of axon growth and neuronal morphogenesis (Fujita and Yamashita, 2014). We therefore performed pull-down assays and observed that S1P treatment of primary DRG neuron cultures activated RhoA within 10 min in a dose-dependent manner (Figures 4A,E). Furthermore, S1P-induced neurite retraction was significantly reduced *in vitro* by the specific Rho inhibitor C3-toxin (Figure 4B; retracted *p* = 0.0005, two sample *t*-test). In addition, the percentage of neurons responding to S1P with an elongation of neurites increased significantly in the presence of C3-toxin (Figure 4B; elongated *p* < 0.001, two sample *t-test*). The same effect was observed with the ROCK inhibitor Y27632, which decreased the percentage of retracted neurons to nearly 30% compared to S1P-treated cultures (Figure 4C; *p* < 0.001, *χ*^2^-test). In contrast, the S1P_1_ receptor selective agonist SEW2871 did not activate RhoA even at high doses (Figures 4D,E), which is in line with the findings obtained in S1P_1_ deficient neurons. Since the effect of S1P on neurite retraction occurred within the first 15 min, we investigated the ROCK-dependent phosphorylation of one of its downstream targets, the CRMP2. CRMP2 binds to tubulin dimers and enhances microtubule formation (Fukata et al., 2002), however phosphorylation at threonine-555 by ROCK, reduces its ability to bind tubulin, leading to microtubule disorganization and growth cone collapse (Arimura et al., 2000, 2005). We assessed phospho-CRMP2 (pCRMP2) levels before and after 15, 30 and 60 min of S1P treatment in primary DRG neuron cultures from adult wt mice. Western blot analysis revealed that S1P induced rapid phosphorylation of CRMP2 at Thr-555, with a 2.6-fold increase at 30 min (Figures 4F,G; ctrl vs. S1P 15’ *p* = 0.019, ctrl vs. S1P 30’ *p* < 0.0001, ctrl vs. S1P 60’ *p* = 0.027, one-way ANOVA followed by Tukey *post hoc* test), while total CRMP2 expression was unchanged (Figure 4H, *p* = 0.2478, one-way ANOVA with multiple comparisons correction). Notably, addition of the ROCK inhibitor, Y27632, inhibited S1P-induced phosphorylation of CRMP2 (Figures 4F,G; *p* < 0.0001, one-way ANOVA followed by Tukey *post hoc* test). Altogether, these results support the hypothesis that S1P is a critical inducer of neurite retraction in adult sensory neurons through S1P_3_ receptor by activating the downstream Rho/ROCK pathway with subsequent ROCK-dependent CRMP2 phosphorylation.

### S1P Produces Retraction of Motor Neuron-Like Cells *in Vitro*

**Figure 5:**
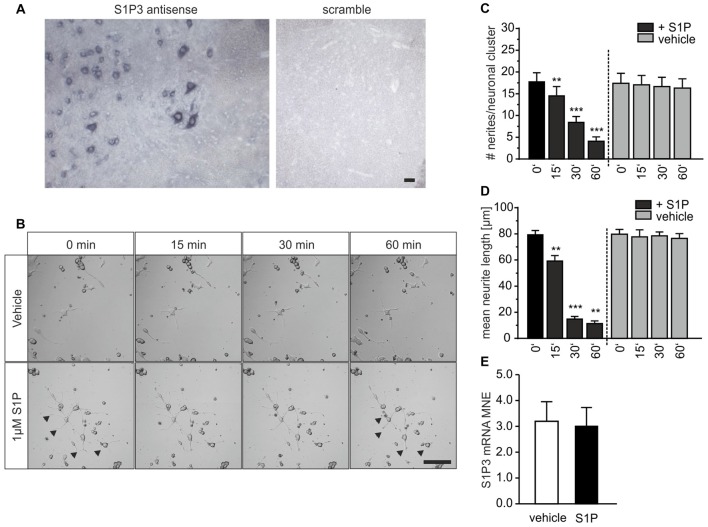
S1P produces retraction of motor neuron-like cells *in vitro*. **(A)** *In situ* hybridization (ISH) experiments in mouse spinal cord sections showed that S1P3 receptor mRNA was expressed in motor neurons. Detection of the S1P_3_ antisense probe showed clear intracellular but not nuclear staining in somata of motor neurons and smaller neurons in the ventral horn. No staining could be detected in the scrambled probe control. Scale bar 100 μm. **(B)** Motor neuron-like cultures were treated with 1 μM S1P for 60 min and observed in a time-lapse experiment. Scale bar 100 μm. **(C)** The number of neurites per neuronal cluster (cells contained within the field of view with clearly identifiable processes) was significantly reduced after S1P stimulation (one-way ANOVA, Bonferroni *post hoc* test; vehicle *n* = 8, S1P *n* = 8). **(D)** After 15 min of S1P also the total neurite length was reduced compared to the vehicle (*p* < 0.001, two-way ANOVA; Tukey *post hoc* test; vehicle *n* = 8, S1P *n* = 8). **(E)** Relative S1P_3_ receptor subtype mRNA expression quantified by qRT-PCR. The target mRNA expression is normalized to endogene expression and amplification efficiency. The mean normalized expression (MNE) for vehicle and S1P treated samples is represented. The S1P_3_ receptor MNE did not differ after S1P treatment (Mann-Whitney test, *P* = 1.000; *n* = 3). **p < 0.01, ***p < 0.001.

S1P_3_ receptor mRNA is expressed in populations of small- and large-diameter DRG sensory neurons as we previously observed (Camprubí-Robles et al., 2013). Since *in situ* hybridization of the spinal cord demonstrated the presence of S1P_3_ receptor mRNA also in motor neurons in the ventral spinal horn (Figure 5A), we investigated whether S1P could affect neuronal outgrowth of motor neurons as for sensory neurons. We cultured motor neuron-like cells for 24 h and exposed them to S1P in a time-lapse experiment. Akin to sensory neurons, motor neuron-like cells started to retract already after 15 min of S1P stimulation, and severely retracted neurites after 30 min (Figure 5B). At 1 μM, S1P treatment significantly reduced the number of neurites per neuronal cluster (Figure 5C; 0 vs. 15 min *p* = 0.0039; 0 vs. 30 min *p* < 0.0001; 0 vs. 60 min *p* < 0.0001, one-way ANOVA, Bonferroni *post hoc* test) and the mean neurite length (Figure 5D; 0 vs. 15 min *p* = 0.0049; 0 vs. 30 min *p* < 0.0001; 0 vs. 60 min *p* < 0.0001, one-way ANOVA, Tukey *post hoc* test) after 30 min stimulation. After 30 min S1P-treated cells had 8 neurites/neuronal cluster compared to 17 neurites/neuronal cluster of the vehicle treated cells. Moreover, S1P treatment decreased the total neurite length of motor neuron-like cells from 79.258 ± 4.202 μm to 59.101 ± 4.192 μm after 30 min (Figure 5D). In line with our *ex vivo* data, motor neuron-like cells significantly expressed S1P_3_ receptor subtype and the normalized mRNA expression did not change after S1P treatment (Figure 5E). Therefore, our data indicate that S1P affects neuronal morphology not only in adult sensory neurons but as well in motor neurons which likewise express the S1P_3_ receptor, indicating a wider action of S1P in functionally different neuron types.

### S1P_3_ Receptor Is Involved in Recovery of Sensory and Motor Function after Peripheral Nerve Injury *in Vitro*

**Figure 6:**
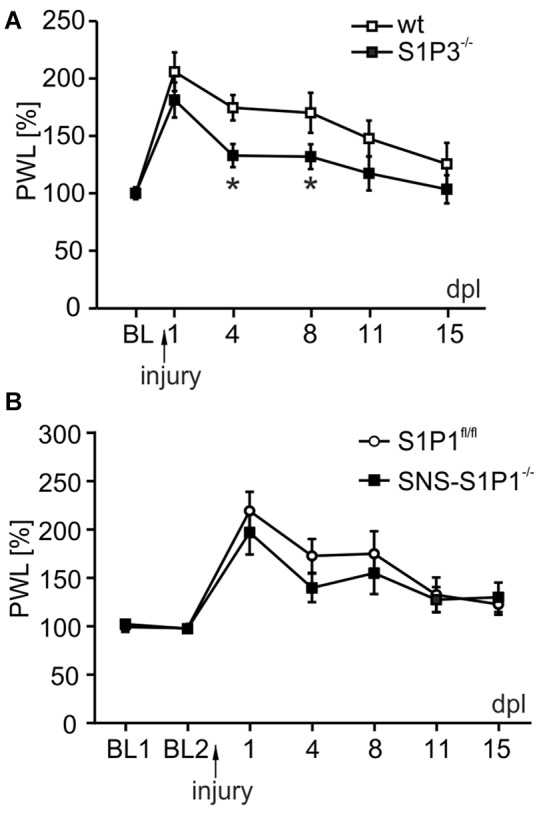
S1P_3_ receptor is involved in recovery of sensory and motor function after peripheral nerve injury *in vivo*. **(A)** S1P_3_^−/−^ animals (black square) underwent sciatic nerve crush and compared to a control group (wt, open square) for sensory thresholds. Already 4 days post-lesion (dpl) S1P_3_^−\−^ mice recovered heat paw withdrawal latencies in comparison to wt animals (174 ± 11% for wt vs. 133 ± 10% for S1P_3_^−\−^; day 4 *p* = 0.016, two way RM ANOVA, Mann-Whitney *post hoc* test with Holm-Bonferroni’s multiple correction; *n* = 10). In wt mice sensitivity returned to baseline only at 15 dpl (day 1 206 ± 17% for wt vs. 181 ± 15% for S1P_3_^−\−^; day 15 125 ± 18% for wt vs. 103 ± 12% for S1P_3_^−\−^), while in S1P_3_^−\−^ animals the recovery was significantly improved from early stages post-lesion (day 1 181 ± 15%; day 4 133 ± 10%; day 8 132 ± 11%, day 8 *p* = 0.045). **(B)** SNS-S1P_1_^−/−^ mice did not show any significant difference in recovery of heat paw withdrawal thresholds compared to control S1P_1_^fl/fl^ mice (two way RM ANOVA, genotype: *F*_(1,131)_ = 0.371, *p* = 0.549; time points: *F*_(6,131)_ = 16.599, *p* < 0.001; genotype × time points: *F*_(6,131)_ = 0.602, *p* = 0.728; *n* = 11). *p < 0.05.

To demonstrate the relevance of S1P_3_ receptor signaling in peripheral nerve regeneration *in vivo*, we used a sciatic nerve crush injury model and monitored sensory thresholds as signature of the functional recovery 15 days post-lesion (dpl). S1P_3_^−\−^ mice recovered significantly faster to normal heat paw withdrawal latencies after crush injury compared to wt control group (Figure 6A; day 4 *p* = 0.016, day 8 *p* = 0.045, two way RM ANOVA, Mann-Whitney *post hoc* test with Holm-Bonferroni’s multiple correction): in both wt and S1P_3_^−\−^ mice heat sensitivity decreased after injury, however remained elevated in wt mice and returned to baseline at 15 dpl (day 1: 206 ± 17% vs. 181 ± 15%, and day 15: 125 ± 18% vs. 103 ± 12% for wt and S1P_3_^−\−^, respectively). In S1P_3_^−\−^ animals the heat sensitivity was significantly improved already from early post-lesion stages (day 1: 181 ± 15%; day 4: 133 ± 10% and day 8: 132 ± 11%). In line with our *in vitro* data, sensory recovery of conditional knock-out animals for S1P_1_ receptor was comparable to wt, indicating that the S1P_1_ receptor is not critically involved in the recovery after peripheral lesion (Figure 6B; two way RM ANOVA, genotype: *F*_(1,131)_ = 0.371, *p* = 0.549; time points: *F*_(6,131)_ = 16.599, *p* < 0.001; genotype × time points: *F*_(6,131)_ = 0.602, *p* = 0.728). These *in vivo* data together with our *in vitro* results introduce S1P_3_ receptor signaling as a general regulator of the outgrowth of peripheral neurons, which appears to be important in the fine-tuning of peripheral nerve regeneration and degeneration.

## Discussion

During neurogenesis, neuronal migration and axonal growth several factors, such as growth factors or chemokines, serve as chemoattractant to guide the neuronal processes towards their target (da Silva and Dotti, 2002). Previous reports have associated S1P with nervous system development and neurogenesis (Brinkmann, 2007; Proia and Hla, 2015). However, the biological function of S1P in the adult nervous system is not fully understood.

In the present study we have explored the role of S1P in axonal outgrowth. We provide *in vitro* and *in vivo* evidence that S1P via S1P_3_ receptors and RhoA/ROCK signaling module induced neurite retraction of adult sensory and motor neuron-like cells which may represent one of the factors hampering neuron regeneration following injury. Moreover, we suggest that S1P-induced neurite retraction might be associated with CRMP2 phosphorylation at threonine-555, which was abolished by ROCK inhibition.

We found that addition of exogenous S1P to the culture medium overnight reduced the number of adult sensory neurons with neurites and their total neurite length *in vitro*, whereas depletion of sphingosine kinase 1 (Sphk1) or pharmacological inhibition of both SphK isoforms 1 and 2 with the ABC294640 inhibitor, to prevent endogenous S1P generation, did not affect the above parameters. This suggests that endogenous neuronal S1P production was not sufficient to account for the observed growth inhibition by S1P, whose role in nerve regeneration is still controversial. Surprisingly, the neurotrophin nerve growth factor NGF, which has a growth promoting activity, causes translocation of SphK1 to the plasma membrane and activation of S1P_1_ and S1P_2_ receptors (Toman et al., 2004). However, S1P also suppresses the NGF-induced neurite extension (Toman et al., 2004) and on Xenopus retinal cells S1P elicits growth cone collapse and repulsive axonal responses (Strochlic et al., 2008).

In view of this dual role of S1P, we studied the effect of S1P on existing neurites. S1P application not only decreased the total neurite length in short-term DRG cultures, but also induced a pronounced retraction of neurites and a collapse of adult sensory neuron growth cones within 30 min.

Since not only sensory but also motor neuron-like cells responded to S1P with a reduction in the number and total length of neurites, it is anticipated that S1P may play a general role in fine-tuning neuronal morphology and axonal outgrowth.

Importantly, S1P-induced retraction was noticeable at 10 nM and saturated at 1 μM S1P. These concentrations are comparable to those found in the plasma (1 μM) and in the lymph (100 nM), and even lower concentrations are measurable in the interstitial fluid of tissues (Proia and Hla, 2015). In *Drosophila* phosphatidic acid phosphatases are involved in generating an extracellular S1P gradient (Zhang et al., 1997) and, in mammals, this S1P gradient regulates cell migration in blood and lymphatic circulatory system (Hla et al., 2008; Aoki et al., 2016). Thus, a possible S1P gradient between the nervous system and the target tissue may be effective to guide growing axons during development or regeneration to correctly polarize and find their targets.

S1P receptors are widely expressed in the nervous system, with S1P_1_ presumably being the most predominant subtype. Adult mouse sensory neurons express the S1P receptor subtypes S1P_1_, S1P_2_ and S1P_3_ (Mair et al., 2011; Wang et al., 2015): S1P_1_ receptors are expressed in the nociceptor population, S1P_2_ receptors in the proprioceptive neurons and S1P_3_ receptors are present virtually in all neurons in the DRG (Mair et al., 2011; Camprubí-Robles et al., 2013). S1P_1_ activation controls cytoskeletal rearrangements and cell motility and enhances cortical actin formation (Rosenfeldt et al., 2001a,b). In line with these reports, we observed that the S1P_1_ receptor agonist rather accelerated neurite outgrowth in short-term adult DRG cultures. The S1P_2_ receptor subtype is involved in cell rounding and neurite retraction in PC12 cells (Toman et al., 2004) and S1P_3_ stimulates cell migration and membrane ruffling in CHO cells (Okamoto et al., 2000). Although S1P_2_ receptors may be associated with anti-regenerative responses upon NOGO binding (Kempf et al., 2014) and be responsible for growth cone collapse in retinal cells (Strochlic et al., 2008), only a negligible fraction of DRG neurons expresses S1P_2_ receptors (Camprubí-Robles et al., 2013), whereas 80% of neurons responded to S1P retracting their neurites. We, for the first time to our knowledge, provide evidence that the retraction induced by S1P was dramatically reduced in S1P_3_ receptor deficient sensory neurons, pointing to an important role of S1P_3_ receptors. Support for this finding comes from different cell lines where S1P_3_ receptor subtype promotes membrane ruffling (Okamoto et al., 2000) and affects cell morphology (Van Brocklyn et al., 1999). These observations have been reported from cell lines, which, however, do not always resemble a neuronal phenotype. S1P binds S1P_1_ and S1P_3_ receptor subtypes with a different affinity (S1P_1_: Kd = 8–20 nM; S1P_3_: Kd = 23–26 nM; Kluk and Hla, 2002; Watters et al., 2011), thus, under physiological conditions supporting axon regeneration after injury, the growth-promoting effect of S1P_1_ receptors might overrule the growth-inhibiting effect of S1P_3_ receptors due to these differences in receptor affinity. Moreover, our data suggest that different concentrations of S1P released at the injury site might fine-tune the growth response during the different phases of axonal regeneration depending on whether S1P_1_ or S1P_3_ receptors are more active. Both growth-promoting and growth-inhibitory factors are released at the injury site during regeneration, as neurotrophic factors, pro-inflammatory and anti-inflammatory cytokines, generating a highly regulated environment to permit regeneration (Gaudet et al., 2011). The finding that S1P still induced neurite retraction in 40% of neurons lacking S1P_3_ receptors indicates a broader scenario and a possible redundancy of the system. Our results on FTY720-P support this complexity and the fine-activation of S1P receptors. FTY720-P is a ligand at S1P_1,3,4,5_ receptors and stimulates axon regeneration after axotomy (Anastasiadou and Knöll, 2016). In sensory neuronal cultures, we observed that FTY720-P induced elongation of neurites rather than retraction like S1P, most likely due to FTY720 activation of S1P_1_ receptors. On the other hand, DRG neurons lacking S1P_1_ receptors showed reduced retraction and enhanced elongation compared to S1P treated neurons. This paradoxical effect could be explained by an antagonistic action at S1P_3_ receptors (Hla and Brinkmann, 2011; Camm et al., 2014). Together, these studies point towards a critical role of S1P signaling in the refined regulation of neuron regeneration, and possibly differentiation, in a cooperative and controlled manner. The fine-tuning between extension and retraction of axons represents an important process during neuroregeneration and synapse formation.

Individual S1P receptors activate different downstream signaling pathways depending on the G proteins which they are coupled with. S1P_1_ receptors are associated with Gi/o and activate Rac to promote migration and vasodilatation, whereas S1P_2_ and S1P_3_ receptors can couple to Gq and G_12/13_ to activate Rho and ROCK and inhibit cell migration and induce vasoconstriction (Okamoto et al., 2000; Brinkmann, 2007). In DRG neuron cultures, RhoA and ROCK were activated upon stimulation with the same S1P dose that promoted retraction, and retraction was abolished by inhibition of RhoA with C3 toxin, while the S1P_1_ receptor agonist did not activate RhoA. Therefore, we explored the involvement of further RhoA effectors. CRMP2 is a member of the collapsin response-mediated protein (CRMP) family and a known target of ROCK phosphorylation (Arimura et al., 2000, 2005). It plays a crucial role in embryonic development, neuronal polarity, in particular in neurite elongation, and is required for axon formation (Wang and Strittmatter, 1996; Inagaki et al., 2001; Arimura et al., 2004). CRMP2 binds to tubulin heterodimers and promotes microtubule assembly. Phosphorylation of CRMP2 inhibits this activity and causes cytoskeleton destabilization (Fukata et al., 2002). A RhoA/ROCK-dependent phosphorylation of CRMP2 at threonine-555 (Thr-555) inactivates CRMP2 and mediates growth cone collapse in DRG neurons (Arimura et al., 2000, 2005). CRMP2 has a prominent role in nerve regeneration after nerve injury (Suzuki et al., 2003) and appears to be involved in Guillain Barrè pathogenesis, where auto-antibodies inhibit neurite outgrowth and axon regeneration *in vivo* and elicit growth cone collapse upon RhoA/ROCK pathway activation and subsequent CRMP2 phosphorylation at Thr-555 (Rozés Salvador et al., 2016). Moreover, motor neurons overexpressing CRMP2 exhibit accelerating reprojection (Suzuki et al., 2003) and inhibition of CRMP2 phosphorylation by cdk5 and GSK3, on sites T509/T514/S518/S522, in a spinal cord injury model results in improved locomotive and nociceptive recovery (Nagai et al., 2016). Our data link ROCK activation by S1P to CRMP2 phosphorylation at Thr-555 for the first time to our knowledge in sensory neurons. CRMP2 phosphorylation was significantly abolished by blocking ROCK activity. S1P binding to S1P_3_ receptors led to retraction of sensory and motor neuron-like cells, and we provide evidence that support the involvement of RhoA/ROCK axis and CRMP2 S1P-dependent phosphorylation in the outgrowth regulation of adult peripheral neurons.

We provide evidence for the relevance of S1P_3_ receptor subtype in S1P-dependent neurite retraction through a transgenic mouse model with a global deletion of S1P_3_, which exhibited improved sensory recovery after nerve crush injury. No such compensation was observed in mice with a sensory neuron specific null mutation of the S1P_1_ receptor subtype.

Based on our present results and previous reports S1P appears to play an important dual role in orchestrating the axonal growth in peripheral neurons, by activation of S1P_1_ receptors for growth-promoting responses and of S1P_3_ receptors for growth inhibition and retraction. Our findings raise the possibility that agonists at the diverse S1P receptors may qualify as promising therapeutic strategies for neurological disorders and neurodegenerative diseases associated with anomalous regenerative responses.

## Author Contributions

SQ, MC-R, DM, RVH and RS carried out the experiments. SQ, MC-R, RVH, RLP, CEB, AF-M and MK designed the study. SQ and MK drafted the manuscript. All authors added to and approved the final manuscript.

## Conflict of Interest Statement

The authors declare that the research was conducted in the absence of any commercial or financial relationships that could be construed as a potential conflict of interest.

## Footnotes

^1^http://www.imagescience.org/meijering/software/neuronj/

## Abbreviations

ABC: ABC294640
CRMP2: collapsin response-mediated protein
dpl: days post-lesion
DRG: dorsal root ganglia
GPCR: G-protein coupled receptor
n.b.c.: neurite bearing cells
NF200: neurofilament 200
NGF: nerve growth factor
PWL: paw withdrawal latency
ROCK: Rho kinase
S1P: sphingosine-1-phosphate
SphK1: sphingosine kinase type 1
wt: wild-type.

## References

[B1] Agarwal N. Pacher P. Tegeder I. Amaya F. Constantin C. E. Brenner G. J.. (2007). Cannabinoids mediate analgesia largely via peripheral type 1 cannabinoid receptors in nociceptors. Nat. Neurosci. 10, 870–879. 10.1038/nn1916 17558404 PMC2234438

[B2] Anastasiadou S. Knöll B. (2016). The multiple sclerosis drug fingolimod (FTY720) stimulates neuronal gene expression, axonal growth and regeneration. Exp. Neurol. 279, 243–260. 10.1016/j.expneurol.2016.03.012 26980486

[B3] Andratsch M. Mair N. Constantin C. E. Scherbakov N. Benetti C. Quarta S.. (2009). A key role for gp130 expressed on peripheral sensory nerves in pathological pain. J. Neurosci. 29, 13473–13483. 10.1523/jneurosci.1822-09.2009 19864560 PMC6664994

[B4] Aoki M. Aoki H. Ramanathan R. Hait N. C. Takabe K. (2016). Sphingosine-1-phosphate signaling in immune cells and inflammation: roles and therapeutic potential. Mediators Inflamm. 2016:8606878. 10.1155/2016/2856829 26966342 PMC4761394

[B5] Arimura N. Inagaki N. Chihara K. Ménager C. Nakamura N. Amano M.. (2000). Phosphorylation of collapsin response mediator protein-2 by Rho-kinase. Evidence for two separate signaling pathways for growth cone collapse. J. Biol. Chem. 275, 23973–23980. 10.1074/jbc.m001032200 10818093

[B6] Arimura N. Menager C. Fukata Y. Kaibuchi K. (2004). Role of CRMP-2 in neuronal polarity. J. Neurobiol. 58, 34–47. 10.1002/neu.10269 14598368

[B7] Arimura N. Menager C. Kawano Y. Yoshimura T. Kawabata S. Hattori A.. (2005). Phosphorylation by Rho kinase regulates CRMP-2 activity in growth cones. Mol. Cell. Biol. 25, 9973–9984. 10.1128/mcb.25.22.9973-9984.2005 16260611 PMC1280267

[B8] Brinkmann V. (2007). Sphingosine 1-phosphate receptors in health and disease: mechanistic insights from gene deletion studies and reverse pharmacology. Pharmacol. Ther. 115, 84–105. 10.1016/j.pharmthera.2007.04.006 17561264

[B9] Camm J. Hla T. Bakshi R. Brinkmann V. (2014). Cardiac and vascular effects of fingolimod: mechanistic basis and clinical implications. Am. Heart J. 168, 632–644. 10.1016/j.ahj.2014.06.028 25440790

[B10] Camprubí-Robles M. Mair N. Andratsch M. Benetti C. Beroukas D. Rukwied R.. (2013). Sphingosine-1-phosphate-induced nociceptor excitation and ongoing pain behavior in mice and humans is largely mediated by S1P3 receptor. J. Neurosci. 33, 2582–2592. 10.1523/jneurosci.4479-12.2013 23392686 PMC6619173

[B11] Cashman N. R. Durham H. D. Blusztajn J. K. Oda K. Tabira T. Shaw I. T.. (1992). Neuroblastoma x spinal cord (NSC) hybrid cell lines resemble developing motor neurons. Dev. Dyn. 194, 209–221. 10.1002/aja.1001940306 1467557

[B12] da Silva J. S. Dotti C. G. (2002). Breaking the neuronal sphere: regulation of the actin cytoskeleton in neuritogenesis. Nat. Rev. Neurosci. 3, 694–704. 10.1038/nrn918 12209118

[B13] Edsall L. C. Pirianov G. G. Spiegel S. (1997). Involvement of sphingosine 1-phosphate in nerve growth factor-mediated neuronal survival and differentiation. J. Neurosci. 17, 6952–6960. 9278531 10.1523/JNEUROSCI.17-18-06952.1997 PMC6573266

[B14] Estrach S. Schmidt S. Diriong S. Penna A. Blangy A. Fort P.. (2002). The human Rho-GEF trio and its target GTPase RhoG are involved in the NGF pathway, leading to neurite outgrowth. Curr. Biol. 12, 307–312. 10.1016/s0960-9822(02)00658-9 11864571

[B15] Fujita Y. Yamashita T. (2014). Axon growth inhibition by RhoA/ROCK in the central nervous system. Front. Neurosci. 8:338. 10.3389/fnins.2014.00338 25374504 PMC4205828

[B16] Fukata Y. Itoh T. J. Kimura T. Ménager C. Nishimura T. Shiromizu T.. (2002). CRMP-2 binds to tubulin heterodimers to promote microtubule assembly. Nat. Cell Biol. 4, 583–591. 10.1038/ncb825 12134159

[B17] Gao P. Peterson Y. K. Smith R. A. Smith C. D. (2012). Characterization of isoenzyme-selective inhibitors of human sphingosine kinases. PLoS One 7:e44543. 10.1371/journal.pone.0044543 22970244 PMC3438171

[B18] Gaudet A. D. Popovich P. G. Ramer M. S. (2011). Wallerian degeneration: gaining perspective on inflammatory events after peripheral nerve injury. J. Neuroinflammation 8:110. 10.1186/1742-2094-8-110 21878126 PMC3180276

[B19] Hargreaves K. Dubner R. Brown F. Flores C. Joris J. (1988). A new and sensitive method for measuring thermal nociception in cutaneous hyperalgesia. Pain 32, 77–88. 10.1016/0304-3959(88)90026-7 3340425

[B20] Hla T. Brinkmann V. (2011). Sphingosine 1-phosphate (S1P): physiology and the effects of S1P receptor modulation. Neurology 76, S3–S8. 10.1212/wnl.0b013e31820d5ec1 21339489

[B21] Hla T. Venkataraman K. Michaud J. (2008). The vascular S1P gradient-cellular sources and biological significance. Biochim. Biophys. Acta 1781, 477–482. 10.1016/j.bbalip.2008.07.003 18674637 PMC2636563

[B22] Inagaki N. Chihara K. Arimura N. Ménager C. Kawano Y. Matsuo N.. (2001). CRMP-2 induces axons in cultured hippocampal neurons. Nat. Neurosci. 4, 781–782. 10.1038/90476 11477421

[B23] Kays J. S. Li C. Nicol G. D. (2012). Expression of sphingosine 1-phosphate receptors in the rat dorsal root ganglia and defined single isolated sensory neurons. Physiol. Genomics 44, 889–901. 10.1152/physiolgenomics.00053.2012 22805346 PMC3472456

[B24] Kempf A. Tews B. Arzt M. E. Weinmann O. Obermair F. J. Pernet V.. (2014). The sphingolipid receptor S1PR2 is a receptor for Nogo-a repressing synaptic plasticity. PLoS Biol. 12:e1001763. 10.1371/journal.pbio.1001763 24453941 PMC3891622

[B25] Kluk M. J. Hla T. (2002). Signaling of sphingosine-1-phosphate via the S1P/EDG-family of G-protein-coupled receptors. Biochim. Biophys. Acta 1582, 72–80. 10.1016/s1388-1981(02)00139-7 12069812

[B27] Li Z. Aizenman C. D. Cline H. T. (2002). Regulation of rho GTPases by crosstalk and neuronal activity *in vivo*. Neuron 33, 741–750. 10.1016/s0896-6273(02)00621-9 11879651

[B26] Li C. Li J. N. Kays J. Guerrero M. Nicol G. D. (2015). Sphingosine 1-phosphate enhances the excitability of rat sensory neurons through activation of sphingosine 1-phosphate receptors 1 and/or 3. J. Neuroinflammation 12:70. 10.1186/s12974-015-0286-8 25880547 PMC4397880

[B28] Mair N. Benetti C. Andratsch M. Leitner M. G. Constantin C. E. Camprubi-Robles M.. (2011). Genetic evidence for involvement of neuronally expressed S1P(1) receptor in nociceptor sensitization and inflammatory pain. PLoS One 6:e17268. 10.1371/journal.pone.0017268 21359147 PMC3040773

[B29] Matusica D. Alfonsi F. Turner B. J. Butler T. J. Shepheard S. R. Rogers M. L.. (2016). Inhibition of motor neuron death *in vitro* and *in vivo* by a p75 neurotrophin receptor intracellular domain fragment. J. Cell Sci. 129, 517–530. 10.1242/jcs.173864 26503157

[B30] Matusica D. Fenech M. P. Rogers M. L. Rush R. A. (2008). Characterization and use of the NSC-34 cell line for study of neurotrophin receptor trafficking. J. Neurosci. Res. 86, 553–565. 10.1002/jnr.21507 17896795

[B31] Meijering E. Jacob M. Sarria J. C. Steiner P. Hirling H. Unser M. (2004). Design and validation of a tool for neurite tracing and analysis in fluorescence microscopy images. Cytometry A 58, 167–176. 10.1002/cyto.a.20022 15057970

[B32] Nagai J. Owada K. Kitamura Y. Goshima Y. Ohshima T. (2016). Inhibition of CRMP2 phosphorylation repairs CNS by regulating neurotrophic and inhibitory responses. Exp. Neurol. 277, 283–295. 10.1016/j.expneurol.2016.01.015 26795088

[B33] Nakamura T. Komiya M. Sone K. Hirose E. Gotoh N. Morii H.. (2002). Grit, a GTPase-activating protein for the Rho family, regulates neurite extension through association with the TrkA receptor and N-Shc and CrkL/Crk adapter molecules. Mol. Cell. Biol. 22, 8721–8734. 10.1128/mcb.22.24.8721-8734.2002 12446789 PMC139861

[B34] Obernosterer G. Martinez J. Alenius M. (2007). Locked nucleic acid-based *in situ* detection of microRNAs in mouse tissue sections. Nat. Protoc. 2, 1508–1514. 10.1038/nprot.2007.153 17571058

[B35] Obinata H. Hla T. (2012). Sphingosine 1-phosphate in coagulation and inflammation. Semin. Immunopathol. 34, 73–91. 10.1007/s00281-011-0287-3 21805322 PMC3237867

[B36] Obreja O. Rathee P. K. Lips K. S. Distler C. Kress M. (2002). IL-1β potentiates heat-activated currents in rat sensory neurons: involvement of IL-1RI, tyrosine kinase, and protein kinase C. FASEB J. 16, 1497–1503. 10.1096/fj.02-0101com 12374772

[B37] Okamoto H. Takuwa N. Yokomizo T. Sugimoto N. Sakurada S. Shigematsu H.. (2000). Inhibitory regulation of Rac activation, membrane ruffling, and cell migration by the G protein-coupled sphingosine-1-phosphate receptor EDG5 but not EDG1 or EDG3. Mol. Cell. Biol. 20, 9247–9261. 10.1128/mcb.20.24.9247-9261.2000 11094076 PMC102182

[B38] Postma F. R. Jalink K. Hengeveld T. Moolenaar W. H. (1996). Sphingosine-1-phosphate rapidly induces Rho-dependent neurite retraction: action through a specific cell surface receptor. EMBO J. 15, 2388–2392. 8665846 PMC450169

[B39] Proia R. L. Hla T. (2015). Emerging biology of sphingosine-1-phosphate: its role in pathogenesis and therapy. J. Clin. Invest. 125, 1379–1387. 10.1172/jci76369 25831442 PMC4409021

[B40] Pyne S. Pyne N. (2000a). Sphingosine 1-phosphate signalling via the endothelial differentiation gene family of G-protein-coupled receptors. Pharmacol. Ther. 88, 115–131. 10.1016/s0163-7258(00)00084-x 11150592

[B41] Pyne S. Pyne N. J. (2000b). Sphingosine 1-phosphate signalling in mammalian cells. Biochem. J. 349, 385–402. 10.1042/0264-6021:3490385 10880336 PMC1221160

[B42] Quarta S. Baeumer B. E. Scherbakov N. Andratsch M. Rose-John S. Dechant G.. (2014). Peripheral nerve regeneration and NGF-dependent neurite outgrowth of adult sensory neurons converge on STAT3 phosphorylation downstream of neuropoietic cytokine receptor gp130. J. Neurosci. 34, 13222–13233. 10.1523/jneurosci.1209-13.2014 25253866 PMC4172810

[B43] Rosenfeldt H. M. Hobson J. P. Maceyka M. Olivera A. Nava V. E. Milstien S.. (2001a). EDG-1 links the PDGF receptor to Src and focal adhesion kinase activation leading to lamellipodia formation and cell migration. FASEB J. 15, 2649–2659. 10.1096/fj.01-0523com 11726541

[B44] Rosenfeldt H. M. Hobson J. P. Milstien S. Spiegel S. (2001b). The sphingosine-1-phosphate receptor EDG-1 is essential for platelet-derived growth factor-induced cell motility. Biochem. Soc. Trans. 29, 836–839. 10.1042/0300-5127:0290836 11709084

[B45] Rozés Salvador V. Heredia F. Berardo A. Palandri A. Wojnacki J. Vivinetto A. L.. (2016). Anti-glycan antibodies halt axon regeneration in a model of Guillain Barr– syndrome axonal neuropathy by inducing microtubule disorganization via RhoA-ROCK-dependent inactivation of CRMP-2. Exp. Neurol. 278, 42–53. 10.1016/j.expneurol.2016.01.016 26804001

[B46] Sanchez T. Hla T. (2004). Structural and functional characteristics of S1P receptors. J. Cell. Biochem. 92, 913–922. 10.1002/jcb.20127 15258915

[B47] Schweigreiter R. Walmsley A. R. Niederöst B. Zimmermann D. R. Oertle T. Casademunt E.. (2004). Versican V2 and the central inhibitory domain of Nogo-A inhibit neurite growth via p75NTR/NgR-independent pathways that converge at RhoA. Mol. Cell. Neurosci. 27, 163–174. 10.1016/j.mcn.2004.06.004 15485772

[B48] Simon P. (2003). Q-Gene: processing quantitative real-time RT-PCR data. Bioinformatics 19, 1439–1440. 10.1093/bioinformatics/btg157 12874059

[B49] Spiegel S. Milstien S. (2002). Sphingosine 1-phosphate, a key cell signaling molecule. J. Biol. Chem. 277, 25851–25854. 10.1074/jbc.r200007200 12011102

[B50] Strochlic L. Dwivedy A. van Horck F. P. Falk J. Holt C. E. (2008). A role for S1P signalling in axon guidance in the *Xenopus* visual system. Development 135, 333–342. 10.1242/dev.009563 18077591 PMC3682207

[B51] Suzuki Y. Nakagomi S. Namikawa K. Kiryu-Seo S. Inagaki N. Kaibuchi K.. (2003). Collapsin response mediator protein-2 accelerates axon regeneration of nerve-injured motor neurons of rat. J. Neurochem. 86, 1042–1050. 10.1046/j.1471-4159.2003.01920.x 12887701

[B52] Toman R. E. Payne S. G. Watterson K. R. Maceyka M. Lee N. H. Milstien S.. (2004). Differential transactivation of sphingosine-1-phosphate receptors modulates NGF-induced neurite extension. J. Cell Biol. 166, 381–392. 10.1083/jcb.200402016 15289497 PMC2172260

[B53] Van Brocklyn J. R. Tu Z. Edsall L. C. Schmidt R. R. Spiegel S. (1999). Sphingosine 1-phosphate-induced cell rounding and neurite retraction are mediated by the G protein-coupled receptor H218. J. Biol. Chem. 274, 4626–4632. 10.1074/jbc.274.8.4626 9988698

[B55] Wang L. H. Strittmatter S. M. (1996). A family of rat CRMP genes is differentially expressed in the nervous system. J. Neurosci. 16, 6197–6207. 8815901 10.1523/JNEUROSCI.16-19-06197.1996 PMC6579169

[B54] Wang J. Wang J. Lu P. Cai Y. Wang Y. Hong L.. (2015). Local delivery of FTY720 in PCL membrane improves SCI functional recovery by reducing reactive astrogliosis. Biomaterials 62, 76–87. 10.1016/j.biomaterials.2015.04.060 26036174

[B56] Watters R. J. Wang H. G. Sung S. S. Loughran T. P. Liu X. (2011). Targeting sphingosine-1-phosphate receptors in cancer. Anticancer Agents Med. Chem. 11, 810–817. 10.2174/187152011797655041 21707490 PMC3698949

[B58] Zhang Y. H. Fehrenbacher J. C. Vasko M. R. Nicol G. D. (2006). Sphingosine-1-phosphate via activation of a G-protein-coupled receptor(s) enhances the excitability of rat sensory neurons. J. Neurophysiol. 96, 1042–1052. 10.1152/jn.00120.2006 16723416

[B57] Zhang N. Zhang J. Purcell K. J. Cheng Y. Howard K. (1997). The Drosophila protein wunen repels migrating germ cells. Nature 385, 64–67. 10.1038/385064a0 8985246

[B59] Zimmermann M. (1983). Ethical guidelines for investigations of experimental pain in conscious animals. Pain 16, 109–110. 10.1016/0304-3959(83)90201-4 6877845

